# Supplementary figures and images for: Multiomic Data Integration in the Analysis of Drought-Responsive Mechanisms in Quercus ilex Seedlings
Source: Plants (Basel). 2022 Nov 12;11(22):3067. doi: 10.3390/plants11223067 (PMC9696786; doi:10.3390/plants11223067)

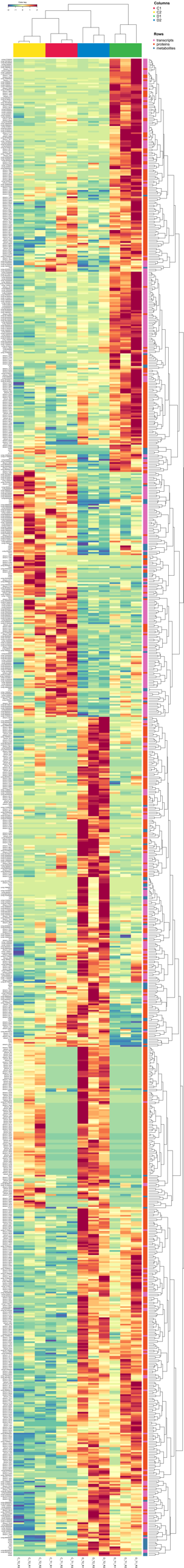

Supplement: Supplementary file 1 [file plants-11-03067-s001.zip › Figure_S1_heapmap_allvariables.pdf]
